# Impact of Maternal Physical Activity and Infant Feeding Practices on Infant Weight Gain and Adiposity

**DOI:** 10.1155/2012/293821

**Published:** 2012-09-26

**Authors:** Lisa Chu, Ravi Retnakaran, Bernard Zinman, Anthony J. G. Hanley, Jill K. Hamilton

**Affiliations:** ^1^The Hospital for Sick Children, Department of Pediatrics, University of Toronto, 555 University Avenue, Toronto, ON, Canada M5G 1X8; ^2^Leadership Sinai Centre for Diabetes, Mount Sinai Hospital, Joseph and Wolf Lebovic Health Complex, 60 Murray Street, Toronto, ON, Canada M5T 3L9; ^3^Division of Endocrinology, University of Toronto, Toronto, ON, Canada; ^4^Samuel Lunenfeld Research Institute, Mount Sinai Hospital, Joseph and Wolf Lebovic Health Complex, 600 University Avenue, Toronto, ON, Canada M5G 1X5; ^5^Department of Nutritional Sciences, University of Toronto, FitzGerald Building, 150 College Street, Toronto, ON, Canada M5S 3E2

## Abstract

Increasing evidence supports the contribution of intrauterine environmental exposures on obesity risk in offspring. Few studies have included maternal and infant lifestyle factors. Our objective was to study the impact of maternal physical activity, infant feeding, and screen time on offspring weight gain and adiposity. In a prospective cohort study, 246 mothers underwent testing during pregnancy to assess glucose tolerance status and insulin sensitivity. Anthropometry and questionnaires on physical activity, infant feeding, and screen time were completed. Multiple-linear regression was performed to examine the impact of maternal and infant factors on infant weight gain and weight-for-length *z*-score at 1 year. Infant weight outcomes were negatively predicted by maternal pregravid vigorous/sport index and exclusive breastfeeding duration. After adjustment, each unit increase in maternal pregravid vigorous/sport index decreased infant weight gain by 218.6 g (*t* = 2.44, *P* = 0.016) and weight-for-length *z*-score by 0.20 (*t* = 2.17, *P* = 0.031). Each month of exclusive breastfeeding reduced infant weight gain by 116.4 g (*t* = 3.97, *P* < 0.001) and weight-for-length *z*-score by 0.08 (*t* = 2.59, *P* = 0.01). Maternal pregravid physical activity and exclusive breastfeeding duration are associated with weight gain and adiposity as early as 1 year of age.

## 1. Introduction

 The increasing prevalence of childhood obesity, especially in preschool aged children [1, 2], has stimulated research targeting critical periods of growth. According to Freinkel's hypothesis of “fuel-mediated teratogenesis,” the intrauterine environment can influence changes in gene expression and affect the development and maturation of fetal organs and tissues [3, 4]. The postnatal period up to 2 years of age is also a critical period of growth [5]. Indeed, rapid infant weight gain is associated with an increased risk of obesity and metabolic consequences later in life [5–8]. 

Emerging evidence suggests that several key factors may influence infant weight gain and adiposity. These include metabolic parameters such as *in utero* glucose exposure [9–11], maternal prepregnancy body mass index (BMI) [12–14], and maternal insulin sensitivity during pregnancy [15]. In a cohort of low-income children, maternal obesity during pregnancy more than doubled the risk of obesity in children [13]. Maternal diet and physical activity during pregnancy [16–19], as well as smoking may impact offspring risk of obesity and metabolic risk [20–23]. However, there is a paucity of literature examining these risk factors in very young children. 

 Infant feeding practices may also modulate later obesity risk. In particular, breastfeeding has a protective role against the development of obesity [24–27]. Harder et al. reported a dose-dependent association between breastfeeding and risk of obesity, where each month of prolonged breastfeeding decreased obesity risk in the child [28]. Beyond the first few months of life, breast-fed infants gained less weight than formula-fed infants [29]. Another study showed weight gain from 6 to 12 months was less in infants exclusively breastfed for ≥5 months compared to ≤2 months [30]. Earlier introduction to complementary foods may also contribute to increased risk of childhood overweight [14, 31], however the evidence is inconsistent [32]. Additionally, reduced physical activity and increased screen time (television and video) are associated with obesity risk in older children, although limited data is available in infants [33, 34]. 

 There is little known about the impact of maternal and infant lifestyle on weight gain and adiposity at 1 year of life. The effect of screen time in infants on weight gain and adiposity has not been included as a potential factor in prior statistical models. There is also little information on the effect of maternal physical activity on weight outcomes of offspring. The objective of our study was to determine how maternal physical activity, maternal insulin sensitivity (IS_OGTT_), prepregnancy BMI, infant feeding practices (breastfeeding duration, age of introduction of formula, and complementary foods), and screen time contribute to infant weight gain and adiposity at 1 year of age.

## 2. Methods

The study protocol was approved by the Research Ethics Board at Mount Sinai Hospital and the Hospital for Sick Children. Study participants consisted of pregnant women attending outpatient obstetrics clinics in Toronto, Ontario. Participants were recruited for this prospective cohort study at the time of antepartum gestational diabetes mellitus (GDM) screening and were consented to be followed into the postpartum. All consenting women then completed a 3-h oral glucose tolerance test (OGTT) in late 2nd or early 3rd trimester. The OGTT was performed in the morning following an overnight fast. Venous blood samples were drawn at baseline, 60, 120, and 180 min after ingestion of a standard 100 g glucose load. The pregnant women were then classified into 3 glucose tolerance groups: (1) GDM, as defined by the National Diabetes Data Group (NDDG) criteria (requires at least two of the following: fasting glucose ≥5.8 mmol/L, 1 h postload glucose ≥10.6 mmol/L, 2 h postload glucose ≥9.2 mmol/L, or 3 h postload glucose ≥8.1 mmol/L); (2) gestational impaired glucose tolerance (GIGT), defined as meeting only one of the above criteria; (3) normal glucose tolerance (NGT), defined as not meeting any of the NDDG criteria [35]. 

 Mothers and infants were asked to attend a followup visit at 1-year postpartum. Parental demographics, medical history and anthropometrics were collected during pregnancy at the time of the OGTT. Data collected included maternal age, maternal prepregnancy weight, maternal and paternal ethnicity, family history of diabetes, socioeconomic status, and maternal physical activity indices. Maternal IS_OGTT_ was calculated from the OGTT using the Matsuda index, which is well correlated with insulin sensitivity derived from the euglycemic-hyperglycemic clamp method [36]. Physical activity was assessed using the Baecke questionnaire, which has been validated in several populations including women of child-bearing age [37, 38]. The questionnaire was completed during the OGTT, with participants reporting on their physical activity in the year preceding the pregnancy. The Baecke questionnaire measures three domains of physical activity: (i) occupation-associated activity (work index); (ii) sport-related physical activity (vigorous/sport index); (iii) leisure-time physical activity not including sports (leisure index). The work index quantifies the exertion related to occupational activities, including sitting, standing, lifting, and walking, as well as effects on the individual (e.g., fatigue and perspiration). The sport index characterizes vigorous/sport activity with respect to intensity (using the updated compendium of physical activities) [39]. These indices were calculated on a scale from 1–5, where 5 represented the highest level of physical activity for each category. For example, a score of 3 out of 5 for leisure index might indicate “sometimes” walking or cycling for 15–30 min and “seldom” watching television. Mean parental education score and occupation score was calculated on a scale of 1–7 and 1–9, based on the Hollingshead index, an established surrogate index of socioeconomic status [40]. A maximum averaged parental score of 62 indicated the highest level of education and profession. 

Infant information collected at birth included length of gestation, gender (male or female), and birth weight. At the 1 year visit, infant weight and length were measured, and weight-for-length *z*-score was calculated according to the World Health Organization (WHO) 2006 child growth standards [41] to provide a surrogate measure of adiposity. The infant lifestyle questionnaire was completed by the mothers when the infants were 3 and 12 months of age and provided information on exclusive breastfeeding duration, age of introduction to formula and cereal, and screen time based on predictors found to be important for the development of obesity (average daily exposure to TV or video viewing, meals eaten while TV is on, TV in bedroom) [34, 42]. Infants born <37 or >42 weeks, twins, or those with medical illnesses that required prolonged hospitalization were excluded from the analysis. 

### 2.1. Statistical Analysis

The statistical analysis was conducted using SPSS version 19.0. Categorical variables are presented as counts and percentages. Continuous variables are presented as mean ± standard deviation. Variables with skewed distributions were logarithmically transformed. Bivariate analysis of continuous variables with infant weight gain and weight-for-length *z*-score were assessed by Spearman's correlation analysis. Independent samples *t*-test or one-way analysis of variance (ANOVA) and post hoc analysis (Fisher's least significant difference) were used to test differences in the outcome variables with maternal glucose tolerance group (GDM, GIGT, or NGT), GDM status (yes/no), family history of diabetes (yes/no), infant gender (male/female), ethnicity (Caucasian or non-Caucasian), age of introduction to formula (at birth, 1–5 months, ≥6 months or never), and average daily screen time divided into 3 categories (no screen time, <60 minutes, and ≥60 minutes). We converted age of introduction to formula into a categorical variable because although exclusive breastfeeding duration and age of introduction to formula are similar variables, they are not synonymous because some infants were never formula-fed (*n* = 80).

Variables determined to be significant from bivariate analyses, concurrently with variables known to influence infant weight gain and adiposity, were entered into multiple linear regression models. Two separate models were created for weight gain from birth to 1 year and weight-for-length *z*-score at 1 year. Nonmodifiable risk factors (infant age, sex, ethnicity, birth weight, and family history of diabetes) were forced into the model and modifiable risk factors (maternal prepregnancy BMI, GDM status, maternal physical activity indices prior to pregnancy, socioeconomic status, maternal log IS_OGTT_, infant exclusive breastfeeding duration, age of introduction to formula, age of introduction to cereal, and daily screen time) were entered according to the forward stepwise method.

## 3. Results

Descriptive characteristics and lifestyle measures are presented in Table 1 for mothers and infants at 1-year postpartum (*n* = 246). From the time of consent postnatally to the 1-year followup visit, 62 (20%) of the original 311 infants were lost to followup. There were no significant differences in GDM status, prepregnancy BMI, maternal education, or maternal physical activity between mothers retained in the study versus those lost to followup.

**Figure 1:**
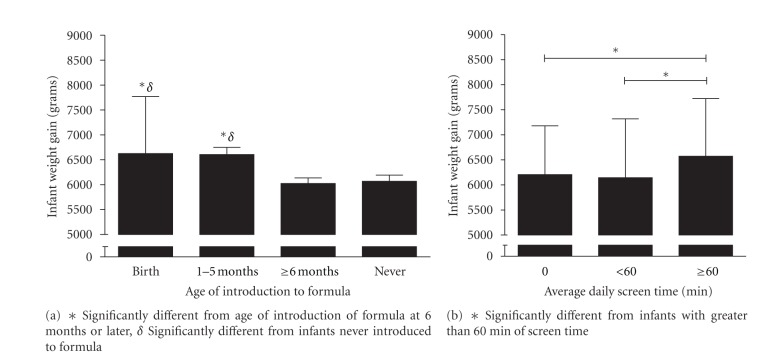
Age of introduction to formula (a) and screen time (b) on infant weight gain at 1 year.

No significant differences were found for infant weight gain and weight-for-length *z*-score based on the mother's GDM status (yes/no) or glucose tolerance group during pregnancy (GDM, GIGT, or NGT). Independent samples *t*-tests also showed no significant differences in the infant outcomes for family history of diabetes (yes/no) and infant ethnicity (Caucasian or non-Caucasian). Males had greater weight gain than females (*P* < 0.001), however no differences between males and females for weight-for-length *z*-score were found (*P* = 0.636). One-way ANOVA showed significant differences in infant weight gain for age of formula introduction (*P* = 0.001) and screen time (*P* = 0.032) (Figure 1). Similar trends for age of formula introduction were found for infant weight-for-length *z*-score at 1 year (*P* = 0.012). However no differences were found between 0, <60 min, or ≥60 minutes of daily screen time (*P* = 0.19).

Spearman's correlation analysis (Table 2) showed a negative association for infant weight gain with maternal pregravid vigorous/sport index (*P* = 0.031), exclusive breastfeeding duration (*P* < 0.001), and an earlier age of introduction to cereal (*P* = 0.02). Weight-for-length *z*-score was positively associated with maternal prepregnancy BMI (*P* = 0.003) and birth weight (*P* < 0.001), and negatively associated with maternal log IS_OGTT_ during pregnancy (*P* = 0.031) and maternal pregravid vigorous/sport index (*P* = 0.006). Maternal pregravid vigorous/sport index was also positively correlated with IS_OGTT_ during pregnancy (*P* < 0.001), as expected from previous data [43].

Results for the multivariate analyses are presented in Table 3. Each month of prolonged exclusive breastfeeding reduced weight gain by 116.4 g, after adjustment for infant age, sex, infant ethnicity, family history of diabetes, and maternal pregravid vigorous/sport index (*P* < 0.001). After adjustment, each unit increase in maternal pregravid vigorous/sport index decreased weight gain by 218.6 g (*P* = 0.016). In total, 33% of the variance in the model for weight gain was explained by the sample. Infant birth weight, infant ethnicity, and family history of diabetes did not significantly predict infant weight gain at 1 year. Additionally, socioeconomic status, maternal prepregnancy BMI, maternal IS_OGTT_, GDM status, maternal pregravid work index, maternal pregravid leisure index, infant age of introduction to formula, and infant screen time did not emerge in the model when entered. For weight-for-length *z*-score, an increase in one unit of maternal prepregnancy BMI (kg/m^2^) was associated with an increase in weight-for-length *z*-score of 0.03 (*P* = 0.016), or one standard deviation change on the WHO child growth standards, after adjustment. Thus, mothers with a greater prepregnancy BMI were more likely to have heavier infants normalized for length. In addition, each month of prolonged exclusive breastfeeding and unit increase in maternal pregravid vigorous/sport index decreased weight-for-length *z*-score by 0.08 (*P* = 0.010) and 0.20 (*P* = 0.031), respectively. Infant sex, infant ethnicity, and family history of diabetes were not significantly associated with infant weight-for-length *z*-score. Variables that did not emerge in the model included: socioeconomic status, maternal IS_OGTT_, GDM status, maternal pregravid work index, maternal pregravid leisure index, infant age of introduction to formula, and infant screen time. Approximately, 19% of the variance in the model for weight-for-length *z*-score was explained by the sample.

## 4. Discussion

 These findings demonstrate that maternal pregravid physical activity, prepregnancy BMI, and infant feeding practices have a significant influence on infant weight gain and adiposity. Weight gain from birth to 1 year was negatively predicted by infant female sex, maternal pregravid vigorous/sport activity, and exclusive breastfeeding duration and positively predicted by infant age at time of 1 year visit. Weight-for-length *z*-score at 1 year was negatively predicted by maternal pregravid vigorous/sport activity and exclusive breastfeeding duration and positively predicted by birth weight, infant age at time of 1 year visit, and maternal prepregnancy BMI. Factors that have been previously shown to influence infant weight gain and weight-for-length *z*-score, such as socioeconomic status or GDM status, were not significant in our model. This may be due to the fact that women in this study tended to have excellent glycemic control, which was evidenced by normal birth weight in the GDM pregnancies. In addition, families tended to be from mid to high socioeconomic status. 

The effect of maternal prepregnancy BMI on infant adiposity is consistent with existing literature [12–14, 31]. In mothers with NGT or GDM, maternal prepregnancy BMI was significantly associated with childhood overweight in both groups, even after adjustment for maternal glucose status and infant birth weight [12]. Furthermore, Knight and colleagues reported that the impact of maternal fasting plasma glucose in nondiabetic mothers on infant growth was transient, while maternal prepregnancy BMI and paternal BMI remained correlated with offspring BMI at 2 years of age. Thus, the authors suggested that parental obesity, shared environment, or genetic factors had a greater influence on childhood BMI than maternal fasting plasma glucose during pregnancy [44]. 

We also found that the maternal environment has an important role on infant growth. After controlling for confounding factors, maternal pregravid vigorous/sport activity significantly predicted infant weight gain. The mean pregravid vigorous/sport index in our study was 2.4, which suggests that 15–30 min of walking or cycling per day [37] in mothers prior to pregnancy may be beneficial for decreasing obesity risk of offspring. Maternal log IS_OGTT_ did not predict infant growth at 1 year, after adjusting for maternal physical activity indices. Prior analyses by our group showed pregravid vigorous/sport activity was an independent predictor of maternal IS_OGTT_ and reduced glucose intolerance in pregnancy [43]. Taken together, pregravid physical activity may affect infant weight gain through a mechanism that partly involves maternal IS_OGTT_, however, these results suggest that maternal lifestyle may have a greater impact on infant weight gain than maternal glucose tolerance group or IS_OGTT_.

Although daily screen time was not a significant predictor of infant weight gain or adiposity in our multivariate analyses, it was unsettling to find that more than half of the infants in our study were already exposed to television at 1 year of age and 11% of infants watched more than 2 hours of television per day. Infants with ≥60 minutes of screen time each day were heavier at 1 year of age than infants with 0 or <60 minutes of daily screen time. One possible explanation may include decreased overall activity levels in infants exposed to television for longer periods of time. However, further examination of infant activity is necessary. In US preschool children, more than 2 hours of television per day was associated with a higher risk of overweight and at risk for overweight and greater adiposity [45]. Despite the known effect of screen time on BMI in childhood [45–47], there is currently limited information on the effect of screen time under 2 years of age. 

The mechanisms to explain the effects of breastfeeding compared to formula feeding may include different physiological and behavioural factors. Several studies [24–27], however not all [32, 48, 49], suggest that breastfeeding has a protective role on later obesity risk. After adjusting for confounding variables, infants exclusively breast fed for a longer duration had reduced weight gain and adiposity at 1 year. This may be influenced by the unique metabolic factors in breast milk compared to formula, such as breast milk leptin [50, 51] and adiponectin [52] that may induce earlier satiety. The higher protein content of formula may also contribute to increased adiposity in formula fed infants. Koletzko and colleagues demonstrated that infants given formula with high protein content compared to infants given formula with low protein content resulted in increased weight at 2 years of age with no effect on infant length [53]. It is thought that the higher protein consumption results in higher insulin secretion and stimulates the expression of insulin-like growth factor I (IGF-I), leading to more adipogenic activity and adipocyte differentiation [54, 55]. Additionally, behavioural differences between breast-fed and formula-fed infants may affect infant energy intake. Studies have shown that there is less maternal control and a greater response to the infant's hunger and satiety cues with breastfeeding compared to formula feeding [56, 57]. There is also a lower frequency of meals and a higher uniformity of feeding volumes in formula-fed infants compared to breast-fed infants [58]. 

Although some studies have reported the timing of cereal introduction as a positive predictor of infant weight gain [14], it did not emerge in our multivariate analyses. In a recent study, Huh and colleagues noted that the timing of the introduction of solid foods on the odds of obesity at 3 years of age varied by breastfeeding status. Infants that were introduced to solid foods before 4 months and were never breast fed or stopped breastfeeding before 4 months had a sixfold increase in the odds of obesity compared to infants breast fed for more than 4 months [59]. Since our study population was introduced to cereal later than 4 months (5.5 ± 1.1 months), this may explain why cereal introduction did not affect weight gain or adiposity. Altogether, our findings on infant feeding suggest that exclusive breastfeeding duration may be more predictive of infant weight gain and adiposity than age of formula or cereal introduction. 

This study should be viewed in context of certain strengths and limitations. Due to the assessment of glucose tolerance in the late 2nd or 3rd trimester of pregnancy, we cannot completely exclude the possibility that some individuals may have received treatments following OGTT that could affect *in utero* glucose exposure and IS_OGTT_ on infant outcomes. Although data was available on the type of intervention (diet therapy or other), glycemic control during pregnancy could not be determined. Other limitations included the lack of data on maternal diet and maternal physical activity during pregnancy in our study. Gluck et al. proposed that maternal energy intake influenced energy intake in children more than intrauterine exposure to diabetes [60], which suggests maternal energy intake should be included in future studies. Additionally, our study population had a large proportion of Caucasian infants and highly educated parents, and it is unclear whether results may be extended to other ethnic backgrounds or families with lower socioeconomic status. Loss to followup was 20%, and although there were no differences in maternal factors between those who left and remained in the study, feeding practices of the infants may have differed. We were also unable to report on infant physical activity due to challenges in capturing physical activity accurately and objectively in infants. Nevertheless, we examined maternal and infant lifestyle variables that have not been previously studied together, providing further insight into the effects of the maternal and infant environment on weight gain and adiposity while controlling for important confounding variables. 

 In summary, increased maternal pregravid physical activity and longer exclusive breastfeeding duration may have a critical influence on reducing infant weight gain and adiposity as early as 1 year of age. Results from this study support the recommendation for exclusive breastfeeding in infancy, and suggest maternal physical activity may also influence postnatal outcomes. Future directions include examining the additive effects of maternal diet during and after pregnancy, infant physical activity, and sedentary time on infant weight gain and adiposity. The evaluation of paternal physical activity may also provide insight into the impact of family lifestyle on infant growth. Longitudinal followup of these children will examine the effects of maternal and infant factors on BMI and adiposity in early childhood beyond 1 year of age.

## Figures and Tables

**Table 1:** Maternal and infant descriptive characteristics (*n* = 246).

		Mean ± SD		*N* (%)
Maternal factors	Age (years)	34.2 ± 4.7	OGTT status	
	Prepregnancy BMI (kg/m^2^)	29.0 ± 5.2	GDM	70 (28.5)
	SES (Hollingshead index)	51.8 ± 8.3	GIGT	38 (15.4)
	Maternal Insulin sensitivity	5.5 ± 3.6	NGT	138 (56.1)
	Pregravid physical activity		Family history of diabetes	146 (59.3)
	Work index	2.4 ± 0.6		
	Vigorous/sport index	2.4 ± 0.8		
	Leisure index	3.1 ± 0.6		
	Physical Activity 1-year postpartum			
	Work index	3.0 ± 0.6		
	Vigorous/Sport Index	2.3 ± 0.8		
	Leisure Index	3.1 ± 0.6		

Infant factors	Birth weight (g)	3438.2 ± 456.8	Age of introduction to formula*	
	Gestation (weeks)	39 ± 1.4	At birth	45 (18.3)
	Age at time of visit (months)	12.4 ± 1.2	1–5 months	55 (22.4)
			After 6 months	62 (25.2)
	Exclusive breastfeeding duration (months)	4.5 ± 2.3	Never	80 (32.5)
	Age of introduction to cereal (months)	5.5 ± 1.1	Females	126 (51.2)
	Daily screen time (min)	48.9 ± 99.8	Caucasian	156 (63.4)
			Screen time	
	Δ Weight from 0-1 year (g)	6312.7 ± 1116.1	None	93 (38)
	Weight-for-length *z*-score	0.23 ± 1.0	<60 min	76 (31)
			60–119 min	48 (20)
			≥120 min	28 (11)

*4 infants were missing information for age of introduction to formula.

SES: socioeconomic status; GDM: gestational diabetes mellitus; GIGT: gestational impaired glucose tolerance; NGT: normal glucose tolerance; Δ Weight: change in weight from 0 to 1 year.

**Table 2:** Spearman's rho correlation for Δ weight from 0-1 year and weight-for-length *z*-score at 1 year.

	Δ Weight (0-1 year)		Weight-for-length *z*-score at 1 year	
	*r*	*P*	*r*	*P*
Maternal age	−0.078	0.224	−0.079	0.223
Socioeconomic status	0.027	0.695	−0.030	0.664
Maternal log IS_OGTT_	−0.070	0.277	−0.139	0.031
Maternal prepregnancy BMI	0.097	0.132	0.189	0.003
Maternal pregravid work index	−0.009	0.896	0.027	0.683
Maternal pregravid vigorous/sport index	−0.139	0.031	−0.177	0.006
Maternal pregravid leisure index	−0.036	0.582	−0.017	0.795
Infant age at time of visit	0.309	**<0.001**	0.116	0.071
Infant birth weight	0.062	0.330	0.302	**<0.001**
Exclusive breastfeeding duration	**−0.234**	**<0.001**	−0.166	**0.012**
Age of introduction to cereal	**−0.150**	**0.020**	−0.060	0.356
Infant screen time	0.100	0.118	0.081	0.207

**Table 3:** Multiple linear regression analysis of dependent variables Δ weight (0-1 year) and weight-for-length *z*-score, respectively.

	*B*	*t*	*P*
Δ Weight (0-1 year)			
Birth weight	−0.01	−0.06	0.950
Infant age at 1 year visit	339.56	5.82	**<0.001**
Infant sex (female)	−648.43	−4.76	**<0.001**
Infant ethnicity	260.53	1.84	0.067
Family history of diabetes	−62.29	−0.45	0.651
Maternal pregravid vigorous/sport index	−218.64	−2.44	0.016
Exclusive breastfeeding duration	−116.37	−3.97	**<0.001**
			*R* ^2^ = 33%
Weight-for-length *z*-score at 1 year			
Birth weight	0.00	3.50	0.001
Infant age at 1 year visit	0.12	2.00	0.047
Infant sex (female)	0.02	0.13	0.894
Infant ethnicity	0.20	1.41	0.160
Family history of diabetes	−0.11	−0.78	0.439
Maternal prepregnancy BMI	0.03	2.43	0.016
Maternal pregravid vigorous/sport index	−0.20	−2.17	0.031
Exclusive breastfeeding duration	−0.08	−2.59	0.010
			*R* ^2^ = 19%

Parameters. (i) Forced: Birth weight, infant age at 1 year visit, infant sex, infant ethnicity (Caucasian versus non-Caucasian), family history of diabetes; (ii) forward stepwise method: socioeconomic status, maternal prepregnancy BMI, Maternal log IS_OGTT_, maternal GDM status during pregnancy (yes/no), maternal pregravid work index, maternal pregravid vigorous/sport index, maternal pre-gravid leisure index, exclusive breastfeeding duration, age of introduction to formula (at birth, before 6 months, after 6 months, never), age of introduction to cereal, and daily screen time.

GDM: gestational diabetes mellitus; IS_OGTT_: oral glucose tolerance testing for assessment of insulin sensitivity.

## Abbreviations

BMI: Body mass index
GDM: Gestational diabetes mellitus
OGTT: Oral glucose tolerance test
NDDG: National Diabetes Data Group
GIGT: Gestational impaired glucose tolerance
NGT: Normal glucose tolerance
IS_OGTT_: Maternal insulin sensitivity.
